# From awareness to action: Reimagining cultural responsiveness in pediatric palliative care

**DOI:** 10.1017/S1478951526102983

**Published:** 2026-07-01

**Authors:** Rizza R. Consad, Rachel-Anne S. Valera

**Affiliations:** Language and Letters Department, Bukidnon State University, Malayblay City, Philippines

To the Editor,

The article “Meta-synthesis of ethnic minority families’ experience of children’s palliative care across developed countries” by Iluno, A., Tatterton, M., and Haith-Cooper, M. (2024) resonates with us because it presents the realities of ethnic minority (EM) families with children in palliative care, but also because we belong to a country, the Philippines, where 9.84 million people or 9.1% of the household population (Philippine Statistics Authority 2023), belong to indigenous groups. In fact, the province where we reside is home to 7 indigenous groups.

The recommendation that the article proposes, which we feel strongly about as language educators, is the need for healthcare providers to improve their cultural sensitivity in communication and attitude. Especially since the healthcare providers deal with families whose children have life-threatening illnesses.

The study reveals that when communication is grounded in respect and cultural awareness, the families feel supported, and healthcare services are more effective. It also shows that EM families in the healthcare context are understood through the lens of cultural awareness and linguistic inclusion.

This study is a reminder that language, culture, and identity are inseparable. Though the study proposes that healthcare providers improve their cultural sensitivity, the next step that may be undertaken is developing a culturally responsive training design that further supports healthcare providers in navigating interactions with EM families in a more informed and context-sensitive manner.

The training design may consider awareness of linguistic diversity, with the basic premise that language is simple, respectful, and culturally appropriate. The development of the design may involve the engagement of the family and elders to include the local knowledge systems in care. These components underscore that communication in palliative care is not only about sharing information clearly, but it also involves understanding how words and contexts are shaped by language and culture (Cain et al. 2018). The design may allow for reflective practice for providers to be aware of their own assumptions and biases.

Developing and testing a culturally grounded training design for healthcare professionals that is flexible and adaptable across different settings may be another research direction after this meta-synthesis study. In doing so, healthcare systems move beyond awareness toward a palliative healthcare that is compassionate, responding to the realities of the minority families because health providers are trained to be linguistically and culturally competent.
